# Understanding cardiac toxicity and immune responses to thoracic radiation therapy in non-small cell lung cancer – implications for future research

**DOI:** 10.3389/fonc.2026.1702343

**Published:** 2026-05-28

**Authors:** Zeta Chow, Ronald Charles McGarry, Jordan Miller, Waleed Fouad Mourad, Ralph Zinner, Bernard Mark Evers, Weisi Yan

**Affiliations:** 1 Department of Radiation Oncology, The Ohio State University, Columbus, OH, United States; 2 Markey Cancer Center, University of Kentucky, Lexington, KY, United States

**Keywords:** heart damage, iOAR, lung radiation therapy, lymphocytopenia, RIHD

## Abstract

Thoracic radiation therapy (RT) remains a mainstay treatment for locally advanced non-small cell lung cancer (NSCLC). However, recent evidence highlights significant long-term toxicities to the cardiac and immune systems, with implications for overall survival. This perspective review article examines the impact of thoracic RT on cardiac substructures and their detrimental correlation with survival. We recognized a discrepancy between major adverse cardiac events and overall survival and formulated an intriguing hypothesis linking thoracic RT to immunosuppression. Radiation to lymphocyte-rich tissues and circulating immune cells may induce profound lymphopenia, compromise immune surveillance, and reduce the efficacy of immunotherapy. We propose a dynamic exchange model between circulating lymphocytes and tumor-infiltrating lymphocytes in the context of thoracic RT and systemic immunotherapy. Herein, we highlight the importance of preserving immune system integrity and incorporating promising immune-sparing techniques in radiation planning. In summary, thoracic RT should be re-envisioned not only as an ablative local therapy, but also as a systemic immune modulator in the management of NSCLC.

## 1 Introduction

Advanced-stage non-small cell lung cancer (NSCLC) remains a disease with poor prognosis. For stage III–IV NSCLC patients, 5-year overall survival (OS) ranges between 10–40%, despite aggressive therapy (1). Amid the development of novel systemic treatments in the past decade, such as immunotherapy, thoracic radiotherapy (RT) remains a mainstay treatment modality for locally advanced NSCLC. The clinical emphasis has traditionally been on achieving disease control, often at the expense of long-term toxicity.

To better elucidate the impact of thoracic RT on cardiac and immunological toxicities, we conducted a focused review of studies investigating the association between cardiac dosimetry and clinical outcomes. This perspective article summarizes the current understanding of cardiac and immune responses to thoracic RT and proposes an intriguing model with directions for future research.

## 2 Current understanding: cardiac toxicity from thoracic radiotherapy as a contributing factor to worse survival

The pivotal phase III RTOG 0617 trial evaluated the efficacy of dose-escalated RT in unresectable stage III NSCLC. Contrary to expectations, patients receiving high-dose RT (74 Gy) exhibited inferior outcomes, with a median OS of 20.3 months compared to 28.7 months in the standard-dose group (60 Gy) (2). Subsequent analyses implicated increased cardiac radiation exposure as a potential contributor to the observed survival decrement. Multivariable modeling revealed that higher radiation dose to the heart was significantly associated with increased mortality (3).

Currently, there is accumulating evidence to suggest that cardiac toxicity compromises long-term outcomes of thoracic RT in treating NSCLC. The mean heart dose (MHD) has been frequently used as a surrogate for cardiac dose and correlated with clinical outcomes. Retrospective studies have demonstrated that higher MHD is associated with detrimental high-grade cardiac events and all-cause mortality (4–6). In addition to MHD, specific cardiac substructures have also been found to be associated with worse outcomes. McWilliam identified that RT dose to the base of the heart was significantly correlated with poor survival (7, 8). Additionally, studies have implicated the left atrium/sinoatrial (SA) node, pulmonary artery (PA), and coronary vessels as critical structures associated with increased cardiac toxicity and detrimental survival (7–12). (**Table 1**) summarizes the sentinel findings of these studies.

**Table 1:** Association between dosimetry measures of global cardiac/substructures and clinical findings.

Study	Cardiac structure	Key dosimetry measures	Key findings
Han (2014) (11)	PA	V45 > 70% V60 > 37%	Worse OS
Dess (2017) (4)	Whole Heart	MHD > 11 Gy	Worse G3+ cardiac events
Stam (2017) (9)	LA SVC	Dmax (median 6.5 Gy EQD2) (LA) D90% (median 0.59 Gy EQD2) (SVC)	Worse non-cancer death
Ma (2017) (10)	PA	V40 > 80% V45 > 68% V50 > 45% V55 > 32%	Worse OS
Vivekanandan (2017) (5)	Whole heart LA	Dmax > 63 Gy (Whole heart) D63Gy > 2.2% (LA)	Worse all-cause mortality
Yegya-Raman (2018) (6)	Whole heart LV/RV LAD	MHD > 20 Gy	Worse symptomatic cardiac events and death
Atkins (2021) (12)	LAD Left circumflex LV	V15 Gy > 10% (LAD) V15 Gy > 14% (Left circumflex) V15 Gy > 1% (LV) Mean coronary artery dose > 7 Gy	Increased risk of MACE and all-cause mortality
McWilliam (2023) (8)	Base (Left coronary artery and SA node)	Median dose: High (10.2 Gy) vs. Low (9.1 Gy)	Worse OS

PA, pulmonary artery; LA, left atria; SVC, superior vena cava; LV, left ventricle; RV, right ventricle; LAD, left anterior descending artery; SA, sinoatrial; MHD, mean heart dose; Dmax, maximal dose; OS, overall survival; MACE, major adverse cardiac events.

### 2.1 Sinoatrial node as a critical substructure in thoracic RT

Anatomically, the SA node resides at the junction of the left atrium, superior vena cava, and ascending aorta—regions subject to fluctuating oxygenation due to dynamic blood flow. McWilliam conducted a voxel-based analysis of RTOG 0617 and identified a critical substructure at the base of the heart, proximate to the SA node and left coronary artery, as it was significantly associated with OS. Dose to this subregion superseded total heart dose in prognostic significance using multivariable analysis (8). An additional retrospective study also found that maximum dose (Dmax) to the SA node was predictive of atrial fibrillation and worse survival (13). Notably, SA Dmax was not associated with other cardiac events or cardiac-specific death, indicating that arrhythmias may affect survival without directly causing death.

### 2.2 Volume-based dosimetry to pulmonary artery as a poor indicator for OS

The PA is a region with high cardiac throughput. It has been hypothesized that continuous RT to the PA trunk, exposing circulating peripheral blood, could dampen the immune cells, as an underlying cause of detrimental clinical outcomes. Two studies have specifically investigated volume-based PA dosimetry and clinical outcomes. Both studies found worse OS with higher volume-based dosimetry to the PA (10, 11).

### 2.3 Dose to coronary artery as a predictive factor for major adverse cardiac events and all-cause mortality

RT to the coronary vessels is thought to cause vascular changes leading to fibrosis, atherosclerosis, and endothelial injuries, thus increasing the risk of symptomatic cardiac events. Yegya-Raman revealed that RT doses to the left ventricle (LV), right ventricle, and left anterior descending artery (LAD) were associated with worse symptomatic cardiac events and mortality (6). A further study by Atkins demonstrated that V15 (percentage of volume receiving at least 15 Gy) in the LV, LAD, and left circumflex artery were predictive of worse outcomes. In addition, a mean coronary artery dose greater than 7 Gy was found to be associated with increased risk of MACE and all-cause mortality (12).

## 3 Observed discrepancies: differences observed in survival in RTOG 0617 cannot be fully explained by cardiac deaths alone

In the landmark RTOG 0617, the 2-year OS was 57.6% for the 60 Gy group compared to 44.6% for the 74 Gy group—a significant 13% absolute reduction in OS. The 5-year OS difference persisted, 32.1% versus 23.0% in the standard-dose and high-dose radiotherapy groups, respectively (2, 3). Whether this difference can be attributed to RT-induced cardiac events alone remains an area of active investigation.

An analysis of the SEER database examined cardiac-specific mortality in stage III NSCLC patients who underwent RT across five time periods from 1988 to 2012 (14). Not surprisingly, as RT techniques improved, cardiac-specific mortality declined. Cardiac deaths represented 3.3% of all deaths within the first year of diagnosis and gradually increased to 10.3% at 5 years. These data suggest that while long-term mortality may be driven in part by cardiac events, short-term differences observed in RTOG 0617 cannot be fully explained by cardiac deaths alone—particularly given that a small fraction of deaths at 2 years were cardiac-related (2). A single-institution study of patients with locally advanced NSCLC treated with thoracic RT reported a 1-year cumulative incidence of MACE and vascular death/nonfatal myocardial infarction of 2.5% and 1.7%, respectively (15). Of 533 deaths observed over a median follow-up of 20 months, only 27 (5.1%) were cardiac-related. This finding mirrors SEER data and further suggests that cardiac death alone cannot account for the marked OS differences in RTOG 0617.

Previous studies have demonstrated a higher risk of grade 3+ cardiac events in patients with pre-existing cardiovascular disease, with a reported 24-month cumulative incidence of 21%. Nevertheless, only 2% of mortality was attributed to cardiac causes (4). A recent meta-analysis of ten studies concluded that while RT is associated with an increased risk of cardiac-specific mortality, cardiac events are not significantly associated with OS in patients with lung cancer receiving RT (16). While one may speculate that cardiac morbidity could be underestimated, the reported mortality attributable to cardiac events remained low. In summary, these studies suggest that additional factors, other than cardiac toxicity, may underlie the reduced survival seen in high-dose RT recipients.

## 4 Generating hypothesis: radiation-induced lymphopenia as a potential cause of worse survival with thoracic radiotherapy

### 4.1 Radiation-induced lymphopenia

Lymphocytes are among the most radiosensitive cells in the human body. Prolonged daily RT, particularly within large vascular volumes or lymphoid-rich tissues, has been shown to induce profound and sustained lymphopenia. *In vitro* studies have shown that 0.5 Gy reduces lymphocyte counts by 10%, while 2 Gy results in a 50% reduction (17). Historical preclinical models, including extracorporeal calf blood irradiation, demonstrated a biphasic response: an initial rapid decline followed by a plateau, suggesting inadequate compensatory lymphopoiesis in lymphoid organs such as lymph nodes and spleen (18).

Indeed, human lymphocytes exposed to radiation under oxygenated conditions demonstrate significantly higher rates of chromosomal aberrations compared to anoxic conditions (19). These cytogenetic alterations—including acentric fragments and dicentric chromosomes—can persist for years post-therapy. Even in the absence of cell death, such damage may compromise lymphocyte function and immunologic competence (20). This raises the possibility that subclinical immune dysfunction, rather than direct cardiac death, may contribute to worsened survival outcomes following high-dose cardiac irradiation.

### 4.2 Mechanisms of radiation-induced immunosuppression

Lymphocytes reside predominantly within lymphoid tissues, the tumor microenvironment, and the circulating blood (21). Radiation exposure to these compartments—either directly within the treatment field or indirectly via peripheral circulation—can result in systemic immunosuppression. Several mechanisms have been proposed, including direct cytotoxicity, delayed immune regeneration, and potential bystander effects (22–24).

Multiple mechanisms contribute to RT-induced immune dysregulation, including lymphocyte apoptosis, T-cell receptor (TCR) mutations, shifts in Th1/Th2 cytokine balance, genomic instability, antigen presentation defects, and perturbation of T-cell homeostasis (25). Radiation-induced T-cell homeostasis disruption may result in a diminished capacity to mount immune responses against new pathogens and latent infections, thereby contributing to increased morbidity and mortality, even when absolute lymphocyte counts appear within normal limits (26). RT has been shown to primarily deplete naive T cells while expanding immunosuppressive regulatory T cells (Tregs), resulting in qualitative changes in immune function rather than simple quantitative deficits (21, 27). Notably, these changes occur without significant loss in antigen-specific CD8+ T-cell frequency or functionality, indicating a shift in immune homeostasis.

Importantly, the immune response to ionizing radiation is not linear. Immune effects vary based on dose, dose rate, radiation quality, and the baseline inflammatory state. Low (<0.1 Gy), intermediate (0.1 Gy–1 Gy), and high (>1 Gy) doses can elicit qualitatively distinct and sometimes biphasic immune responses (28). Experimental models suggest that low/intermediate RT may trigger shifts from pro-inflammatory (Th1) to anti-inflammatory (Th2) immune profiles and accelerate immune aging (29). At low doses, surviving T cells may undergo phenotypic changes consistent with immune senescence, likely driven by radiation-induced oxidative stress, DNA damage, chromosomal aberrations, and telomere shortening (30, 31). Studies have shown that irradiated T cells die primarily via apoptosis, with secondary necrosis playing a significant role at higher doses (0.5–2 Gy) (32, 33). Notably, secondary necrotic cells—while non-functional—may still be detected in routine lymphocyte counts, potentially underestimating the extent of immune impairment.

Ilienko studied 235 Chernobyl clean-up workers and found that low-dose RT was associated with reduced CD4+/CD8+ ratios, increased Tregs, and elevated IL-1β levels—features suggestive of immune dysfunction (34). A separate cohort analysis reported that T-cell profile alterations preceded cancer onset by 1–3 years (35). These findings raise the possibility that low-dose RT may contribute to early immunologic changes relevant to tumor surveillance.

### 4.3 Clinical consequences

The lethal dose to reduce circulating lymphocyte populations by 90% (LD90) is approximately 3 Gy. Yovino et al. reported that during standard glioblastoma RT (2 Gy × 30 fractions), over 95% of circulating blood cells cumulatively receive >0.5 Gy, sufficient to induce widespread lymphocyte apoptosis (18). Post-treatment lymphopenia has been associated with inferior tumor control and survival across multiple malignancies, including glioblastoma, head and neck squamous cell carcinoma, cervical, esophageal, lung, and pancreatic cancers (36).

Radiation dose to circulating immune cells has been quantified using the effective dose to immune cells (EDIC), which correlated strongly with severe lymphopenia and poor outcomes, including OS, progression-free survival, and distant metastasis-free survival (37). Specifically, EDIC >4 Gy was associated with particularly adverse effects. Tang et al. confirmed that larger tumor volume was associated with greater lymphocyte depletion and worse survival in NSCLC, though the exact biological mechanisms remain incompletely understood (38).

## 5 Connecting the dots: immunosuppression induced by in-field radiation to circulating lymphocytes as an attributable cause of worse survival

A significant proportion of circulating lymphocytes were directly exposed to radiation during thoracic RT. Morisada et al. demonstrated that the proportion of peripheral blood lymphocytes exhibiting more than six γ-H2AX foci per cell increased from 2.5% to 10% within one hour post-irradiation (39). This finding indicates that a significant portion of circulating lymphocytes passed through the radiation field and remained in the peripheral circulation at the time of sample collection. Given that blood sampling occurred only one hour post-irradiation, and that lymphocyte transit through lymph nodes typically requires approximately 10 hours (40, 41), it is unlikely that the γ-H2AX-positive cells originated from recirculation through lymphoid tissues. Instead, they must have been present in the systemic circulation at the time of irradiation. These findings challenge the conventional assumption that lymphocytes spend minimal time in peripheral blood and underscore the potential for substantial radiation exposure to circulating immune cells, particularly in high-flow regions. Furthermore, the increase in γ-H2AX foci positively correlated with mean lung dose, reinforcing the concept that thoracic RT, due to the high vascular throughput, significantly contributes to systemic immune cell exposure (39). This observation suggests that the radiation dose to regions with high vascular throughput may play an under-recognized role in driving post-treatment lymphopenia.

Treatment of large target volumes, especially those encompassing secondary lymphoid organs such as the spleen, has also been associated with a measurable decline in systemic immune function. While tumor-specific lymphocytes represent only a minor fraction of the circulating lymphocyte pool, lymphopenia has nonetheless been correlated with inferior OS in multiple cancers (42, 43). This supports the hypothesis that circulating immune cells play a critical role in antitumor immunity or, alternatively, reflect the functional status of the immune system (23).

The EDIC model was previously found to be significantly and independently associated with OS and local progression-free survival (44). To build upon this knowledge, we propose a model of dynamic exchange between circulating lymphocytes and tumor-infiltrating lymphocytes (TILs), wherein peripheral T cells may be recruited to the tumor site to replace exhausted or radiation-depleted TILs. Supporting this, a recent study in NSCLC brain metastases treated with stereotactic radiosurgery demonstrated near-complete depletion of intratumoral CD8+ T cells, followed by repopulation with circulating T-cell clones—most of which lacked tumor specificity (45). This suggests that systemic immune cells may be essential for maintaining immune surveillance following localized radiation injury to the tumor microenvironment.

## 6 Application of immune-sparing radiation in clinical practice

Radiotherapy has traditionally served as a localized, cytotoxic modality for cancer treatment. However, in the context of immunotherapy, an evolving paradigm has emerged in which RT is conceptualized as an immunomodulatory modality. This prompts a re-evaluation of the longstanding principle of ablative therapy in treating NSCLC.

### 6.1 Shift towards hypofractionation

Hypofractionation has demonstrated oncologic equivalence to conventional fractionation while offering potential immune benefits. Fewer fractions reduce cumulative exposure of circulating lymphocytes. Hypofractionated RT has been associated with a lower risk of severe lymphopenia and improved survival in both unresectable stage III NSCLC and breast cancer (46, 47). According to the EDIC equation, lowering the number of fractions directly reduces radiation exposure to immune cells (48, 49).

### 6.2 Redefine target volume delineation

Advancements in image-guided RT and motion management have effectively reduced planning target volumes. Stereotactic centralized ablative radiation therapy differentially delivers a high dose to the hypoxic tumor core and a lower dose to the periphery, allowing sparing of abutting lymphoid tissues. It is hypothesized that ablative doses in the tumor core may trigger immune priming through endothelial damage and antigen release, whereas low-dose radiation at tumor margins could activate interferon pathways and CD8+ T-cell recruitment (50, 51). Partial tumor irradiation techniques, such as spatially fractionated radiotherapy and partial tumor irradiation targeting hypoxic segments, reduce target volumes and similarly spare immune cells. With careful target delineation and treatment planning, one can leverage the immune response to potentially achieve better outcomes.

### 6.3 Improve radiation technology to minimize low-dose scatter

To further limit lymphocyte exposure, highly conformal RT techniques must evolve to minimize scattered radiation and delivery time. Proton therapy can be utilized to reduce MHD compared to intensity-modulated photon therapy. In photon-based RT, flattening filter-free beams provide sharp dose fall-off and reduce treatment times, thereby decreasing exposure to adjacent lymphoid-rich tissues and circulating peripheral blood (52). Furthermore, fast linear accelerator-based high-dose-rate radiation therapy could significantly reduce delivery time, and thus minimize exposure to circulating peripheral blood (53). Accordingly, these techniques may have merit in further preserving immune integrity.

### 6.4 Incorporate immune organs at risk

**Figure 1:**
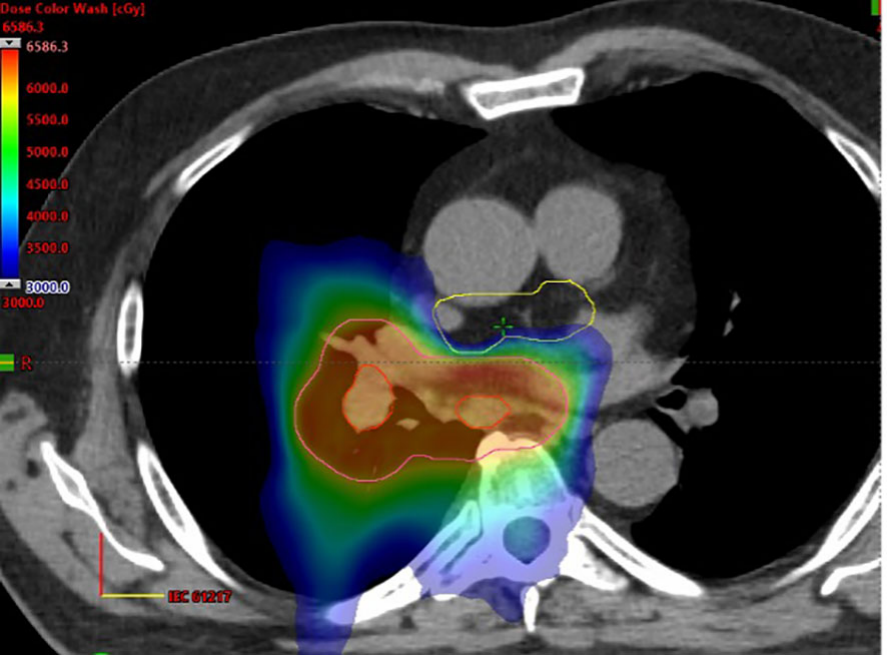
Avoidance of high-dose radiation to pre-defined iOAR. The base of heart, encompassing SA node and origin of LAD, is noted in yellow line. The gross target volume is noted in red line. The planning target volume is noted in orange line. The color-washed region denotes region receiving at least 30 Gy with a global max up to 6586 cGy.

Current RT planning does not systematically account for lymphocyte-rich structures such as uninvolved lymph nodes, spleen, thymus, bone marrow, and major vascular structures. The existing EDIC model represents a framework but is limited by oversimplified assumptions and a lack of spatial specificity (54). Therefore, it is crucial to designate immune organs at risk (iOARs) as avoidance structures during treatment planning. For instance, McWilliam previously identified a cardiac substructure at the base of the heart, a region subjected to high cardiac throughput, to be more prognostic than MHD (7). (**Figure 1**) demonstrates volumetric arc radiotherapy to the planning target volume with deliberate avoidance of high-dose radiation to the base of the heart, designated as an iOAR, in treating a locally advanced-stage NSCLC patient.

### 6.5 Modeling and AI integration

To refine iOAR dosimetry, analytical and Monte Carlo models are under development to estimate out-of-field radiation. AI-based planning tools have been proposed to simulate lymphocyte trafficking and immune dynamics in real time, using Markov chains to model dose distributions across the immune compartment. These predictive models would enable treatment planning systems to optimize immune preservation while maintaining tumor control (55).

## 7 Controversy regarding immunotherapy and its application

The role of the immune system in the genesis and control of cancer remains controversial. With some exceptions, most cancers may not express antigens that are recognized as foreign by classical immune mechanisms. Immunosuppressed individuals develop excess cancers, suggesting that there is an endogenous mechanism to control early cancers. It is also thought that these systems begin to break down with age and exposure to carcinogens, such that cancers may progress. The Goldie–Coldman hypothesis is a mathematical model predicting that tumor cells are intrinsically genetically unstable, producing chemotherapy-resistant phenotypes of cancer cells (56). The corollary to this is the production of metastatic variants and, potentially, immune-resistant clones. Complicating this further is the concept that the immune system may be overwhelmed by rapidly growing cancer cells that invoke immunosuppression through mechanisms such as programmed death ligands.

The formation of cancer nodules and masses appears to reflect the biology of early tumor development, in which daughter cells have a high death rate but selection gradually favors surviving clones. As masses develop, we postulate that an immunologically privileged microenvironment may form within nodules and masses, limiting immune-mediated killing of cancer cells through poor microvasculature and perhaps hypoxia. The question then arises whether non-ablative doses of radiation will improve the effectiveness of immunotherapy, particularly given the concept of repopulation. By administering these non-ablative doses of radiation, one could be inducing more rapid growth of cancer masses by any or all of these mechanisms (57). One would also need to postulate that a defined antigen recognized by the immune system is released through radiation-induced destruction of some cancer cells. With exceptions, most cancers are “self” and are probably not recognized in a standard immune response. Clearly, our understanding of the complexities of local microenvironments in cancer masses is relatively limited, but trials of non-ablative RT are certainly warranted, albeit with appropriate skepticism.

Although this mini-review focuses on locally advanced NSCLC, the proposed framework of cardiac and immune preservation may extend to other malignancies. Treatment-related lymphopenia and EDIC have been associated with inferior outcomes across multiple disease sites, including glioblastoma, head and neck, cervical, esophageal, breast, and pancreatic cancers, indicating that radiation exposure to circulating lymphocytes and lymphoid-rich structures is a general phenomenon rather than one specific to lung cancer. Nevertheless, the NSCLC population is distinct from, for example, early-stage breast cancer: patients with lung cancer are typically older, more likely to have a heavy smoking history and pre-existing cardiopulmonary disease, receive larger thoracic target volumes with higher cardiac and pulmonary artery doses, and are frequently treated with concurrent chemoradiation followed by immune checkpoint blockade. In contrast, most breast radiotherapy is delivered in smaller fields, often in younger patients with fewer comorbidities and without routine immunotherapy. These differences likely magnify the impact of cardiac and immune toxicity in NSCLC and justify our emphasis on this population. Future work should determine, in a disease-specific manner, how immune-sparing planning strategies and iOAR constraints can be optimized for breast and other cancers.

## 8 Conclusion

A conceptual shift is emerging wherein radiation is viewed not solely as an ablative local therapy, but as a biologically active agent and an integral component of precision medicine. In this manuscript, we have examined thoracic radiation toxicity through the lens of immune system preservation and its implications for oncologic outcomes. We also outlined future directions for immune-priming radiotherapy to both enhance local tumor immunogenicity and exert systemic immune-protective effects when appropriately implemented.

Ongoing large-scale efforts, such as the LySAIRI project, are laying the groundwork for the integration of lymphocyte-sparing radiotherapy into routine clinical practice (46). When delivered with anatomical specificity and immunologic intent, radiation has the potential to simultaneously control local disease and modulate systemic immunity. Strategic targeting of intratumoral and peritumoral regions, with consideration for immune system dynamics, aligns with a holistic therapeutic approach and may ultimately improve clinical outcomes. These initiatives aim to harmonize advanced radiotherapy techniques with immunotherapeutic strategies, thereby maximizing tumor control while preserving immune competence. As this field evolves, it is imperative that both clinical protocols and radiation planning paradigms adapt to fully realize the synergistic potential of radio-immunotherapy.
